# Preoperative quality of life in patients undergoing liver surgery

**DOI:** 10.1016/j.sopen.2026.06.008

**Published:** 2026-07-02

**Authors:** Carlos Constantin Otto, Anna Mantas, Alexander Genchev, Aleksandar V. Kargaliev, Amal Mugdad, Smiths Sengkwawoh Lueong, Anna Ewa Cyrek, Florian W.R. Vondran, Ulf P. Neumann, Jan Bednarsch

**Affiliations:** a Department of Surgery and Transplantation, University Hospital Essen, Essen, Germany; b Department of Surgery and Transplantation, University Hospital RWTH Aachen, Aachen, Germany; c German Cancer Consortium (DKTK), partner site Essen, a partnership between German Cancer Research Center (DKFZ) and University Hospital Essen, Essen, Germany; d Bridge Institute of Experimental Tumor Therapy (BIT) and Division of Solid Tumor Translational Oncology (DKTK), West German Cancer Center, University Hospital Essen, University of Duisburg-Essen, Essen, Germany; e Department of Surgery, Maastricht University Medical Center (MUMC), Maastricht, Netherlands

**Keywords:** Quality of life, Liver surgery

## Abstract

**Background:**

Surgical resection remains a cornerstone in the treatment of various hepatobiliary diseases. However, preoperative quality of life (QoL) in patients undergoing liver resection remains insufficiently studied. This study aimed to evaluate preoperative QoL and identify factors associated with impaired QoL in patients scheduled for liver surgery.

**Methods:**

This single-center prospective observational study included patients undergoing liver resection for hepatobiliary diseases. Preoperative QoL was assessed using the Short Form-36 (SF-36) Health Survey, comprising eight domains and the Physical and Mental Component Summary (PCS and MCS) scores. Scores were compared with reference values from the 1998 German Federal Health Survey. Univariate and multivariable linear regression analyses were performed to identify preoperative predictors of QoL.

**Results:**

159 patients were included in the final analysis. Of these, 20 (12.6%) had benign lesions, 80 (50.3%) primary malignant tumors, and 59 (37.1%) secondary malignant tumors. Compared with the general German population, patients demonstrated significantly lower scores in 7 of 8 SF-36 domains as well as in both summary scores. Multivariable analyses identified female sex, absence of preoperative chemotherapy, anemia, history of depression, cardiovascular disease, and renal disease as independent predictors of impaired QoL across multiple domains. The mean R^2^ across all multivariable models was 0.23.

**Conclusions:**

Patients awaiting liver resection exhibit substantially impaired QoL compared with the general population. A combination of demographic, clinical, and laboratory factors influences preoperative QoL. Recognition of these factors may facilitate risk stratification, improve preoperative counseling, and support targeted interventions for patients at increased risk of impaired QoL.

## Graphical abstract

**Figure:**
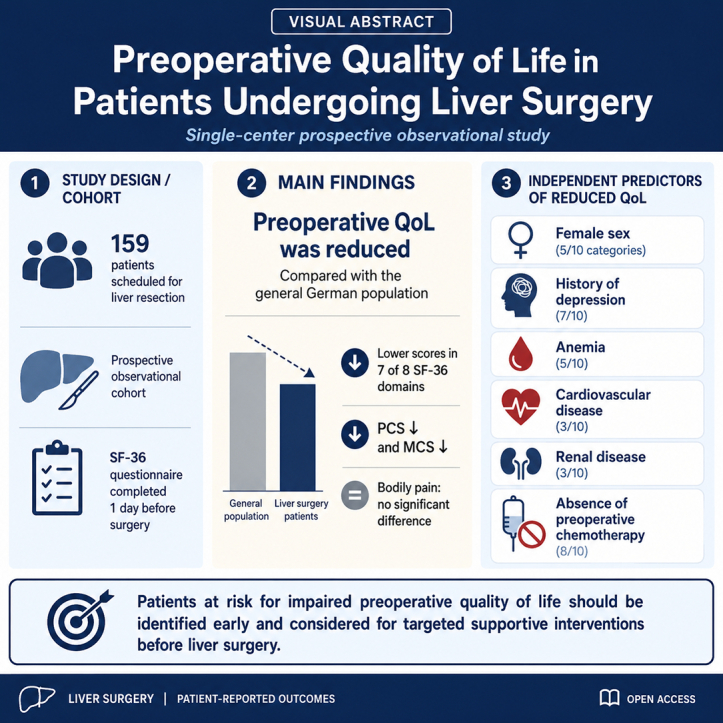

## Introduction

Liver resection displays an important instrument in the treatment of various liver diseases, commonly primary and secondary malignant lesions as well as benign liver tumors, e.g. hepatocellular adenoma, symptomatic hemangioma or focal nodular hyperplasia [1]. Patients subjected to liver resection might experience symptoms of the underlying disease and the preoperative treatment such as physical discomfort, pain, weight loss or adynamia among others [2]. Such symptoms are often closely related to aspects of health-related Quality of Life (QoL). QoL is a multilayered term for patients´ social, physical as well as emotional functionality and role function. In recent years, QoL gains recognition as equally important as other post-therapeutic outcomes e.g. postoperative morbidity or long-term oncological survival [3]. Over the past decades, advancements in surgical techniques and perioperative management have broadened the indications for liver resection highlighting the importance of a holistic outcome assessment in these patients. Also, the further widespread of radiologic imaging technologies increased the incidents of benign liver tumors leading to more surgical procedures in these particular patients [4].

While perioperative QoL has been extensively studied in the context of living donor liver transplantation over the past two decades [5], [6], data on QoL in patients undergoing liver resection —particularly outside of the Asian context where hepatocellular carcinoma (HCC) is more prevalent—remain limited [7]. Most existing studies have focused on postoperative outcomes, with less attention paid to the preoperative period. Here, we aimed to assess preoperative QoL in a large European cohort of a hepatobiliary center with indication for liver resection to identify individuals at risk for impaired QoL using the Short Form 36 (SF-36) Health Survey, a widely used and validated instrument capable of reliably assessing both physical and mental health domains.

## Material and methods

### Patients

Patients scheduled for liver resection in 2022 were consecutively included in this prospective observational study. Patients who finally underwent other treatments than operative resection were excluded from the data set. This study was conducted in concordance with the requirements of the Institutional Review Board (EK177-21), current version of the Declaration of Helsinki and the good clinical practice guidelines (ICH-GCP). Informed consent was obtained from all included patients. The full official name of Institutional Review Board in Aachen is the Independent Ethics Committee of the Faculty of Medicine at RWTH Aachen University.

### Indication, staging, clinical work-up

All patients in this cohort underwent a comprehensive preoperative evaluation tailored to their specific indications. Cross-sectional imaging with either CT or MRI was performed in all cases. Additional diagnostic procedures, such as ERCP or MRCP, were conducted based on the underlying etiology (e.g., perihilar cholangiocarcinoma) and further evaluated as previously described [8], [9]. In cases of malignant neoplasms, surgical decisions were made in accordance with recommendations from the interdisciplinary tumor board. The indication for each surgical procedure was determined by an experienced hepatobiliary surgeon. Patient physical status was assessed using the American Society of Anesthesiologists (ASA) classification. A history of depression was defined as a previously established clinical diagnosis documented in the medical records, including discharge letters or outpatient reports, and/or ongoing antidepressant treatment at the time of preoperative evaluation. No patient-reported screening questionnaires were used to define depression.

### Quality of life

Patients' quality of life (QoL) was assessed using the SF-36 questionnaire. The SF-36 questionnaire was self-administered by all patients without assistance one day prior to surgery. The detailed structure of the instrument has been thoroughly described in the existing literature [10]. Originally, the SF-36 Health Survey has been published and validated by Ware et al. [11]. The questionnaire consists of multiple items grouped into eight domains: physical functioning (PF), role-physical (RP), bodily pain (BP), general health (GH), vitality (V), social functioning (SF), role-emotional (RE), and mental health (MH). These are further summarized into two composite scores: the Physical Component Summary (PCS) and the Mental Component Summary (MCS). Each domain and summary score ranges from 0 to 100, with higher scores indicating better QoL. In general, patients completed the questionnaire one day prior to surgery. The results were calculated using HTS 5 software and compared with reference values from a representative German population sample collected during the 1998 German Federal Health Survey and published by Ellert and Bellach (Hogrefe, Germany [12]). Since individual-level reference data were not available to us, no age- or sex-matching with the reference population was performed.

### Statistical analysis

Categorial data are presented in absolute number with percentages and continuous variables median with interquartile range. Primary endpoint of the study was the association between preoperative parameters with QoL of the SF-36 scales. Therefore, separate univariate and multivariable linear regression models were carried out with various preoperative parameters and the 8 domains of QoL and 2 summary scales, respectively. In here, nominal and categorical data were recoded into a scaled dummy variable. The secondary endpoint was general comparison between QoL of the cohort and a general German population [12]. For better comparison, in here QoL scores are presented with mean and standard deviation, respectively. *P* ≤ 0.05 was set as significant. SPSS Statistics 24 was used for statistical analyses (IBM Corp., Armonk, NY, USA).

## Results

### Patient cohort

The number of recruited patients for this study was 193 of which 17 (8.8%) finally received a non-surgical treatment and 17 (8.8%) returned an incomplete questionnaire resulting in exclusion from the assessable cohort. Finally, 159 patients (82.4%) were included for analysis. The cohort consisted of 87 male (54.7%) and 72 female (45.3%) patients with a median age of 62 years (55–72). Most patients had an ASA score below III (94 patients, 59.1%). Notably, 70 patients (44.0%) had a history of cardiovascular disease (CVD), and 19 (11.9%) had been diagnosed with a depressive disorder. The main indication for liver resection were colorectal liver metastasis in 55 cases (34.6%), followed by cholangiocarcinoma with 52 (32.7%) and hepatocellular carcinoma in 24 cases (15.1%). A subset of 21 patients (10.9%) presented with a benign liver lesion. A considerable proportion of the cohort underwent preoperative systemic chemotherapy (72 patients, 45.3%). Preoperative liver function was generally well-compensated, with a median INR of 1.00 (range: 0.95–1.07), a bilirubin level of 0.49 mg/dL (range: 0.32–0.78), and a creatinine level of 0.84 mg/dL (range: 0.70–0.98). The median hemoglobin concentration was 13.1 g/dL (range: 11.8–14.1). Most frequent extent of liver resection was atypical resection (63/159, 39.6%), followed by left or right hepatectomy in 42 (26.4%) and extended hepatectomy in 20 cases (12.5%). From a social perspective, more than half of the cohort was both married (108/159, 67.9%) and retired or unemployed (83/159 cases, 52.2%). Further details are presented in Table 1.

**Table 1:** Study cohort.

Variables	Cohort (*n* = 159)
Demographics	
Sex, m/f (%)	87 (54.7)/72 (45.3)
Age (years)	62 (55–72)
BMI (kg/m^2^)	26.4 (23.5–29.6)
Preoperative systemic chemotherapy	72 (45.3)
Diabetes mellitus, n (%)	29 (18.2)
Cardiovascular disease, n (%)	70 (44)
Pulmonary disease, n (%)	28 (17.6)
Chronic kidney disease, n (%)	10 (6.3)
ASA, n (%)	
I	6 (3.8)
II	88 (55.3)
III	65 (40.9)
IV	0
Indication for liver resection, n (%)	
HCC	24 (15.1)
CCC	52 (32.7)
CRLM	55 (34.6)
Metastasis by other primary	6 (3.7)
Adenoma	2 (1.3)
FNH	3 (1.9)
Hemangioma	5 (3.1)
Cyst (benign)	6 (3.8)
Others (no malignant lesion)	5 (3.1)

Preoperative liver function	
AST (U/l)	34 (25–55)
ALT (U/l)	27 (18–43)
GGT (U/l)	73 (33−202)
Total bilirubin (mg/dl)	0.49 (0.32–0.78)
Platelet count (/nl)	248 (193–317)
INR	1 (0.95–1.07)
Quick, %	98 (87–107)
Creatinine (mg/dl)	0.84 (0.7–0.98)
Hemoglobin (g/dl)	13.1 (11.8–14.1)

Preoperative quality of life measurements	
Physically functionality	75 (50–90)
Physically role function	25 (0−100)
Physically pain	74 (41–100)
General health perception	52 (42–67)
Vitality	55 (35–65)
Social role function	62.5 (50–100)
Emotional role function	66.7 (0–100)
Mental well-being	68 (48–80)
Physical SF-36 score	43.6 (34.5–51.4)
Mental SF-36 score	45.4 (32.2–52.8)

Operative data	
Laparoscopic resection, n (%)	62 (39)
Conversation rate, n (%)	12 (7.5)
Operative time (minutes)	232 (180–330)
Operative procedure, n (%)	
Atypical	63 (39.6)
Segmentectomy	9 (5.7)
Bisegmentectomy	8 (5)
Hemihepatectomy	42 (26.4)
Extended hemihepatectomy	20 (12.5)
ALPPS/TSH	8 (5)
Central liver resection	2 (1.3)
Other	7 (1.3)
Additional procedures, n (%)	28 (17.6)
Pringle maneuver, n (%)	6 (3.8)
Intraoperative blood transfusion, n (%)	23 (14.5)
Intraoperative FFP, n (%)	11 (6.9)

Social status	
Marital status, n (%)	
Married/partnership	108 (67.9)
Divorced	14 (8.8)
Widowed	14 (8.8)
Unmarried	11 (6.9)
n/a	12 (7.5)
Child(ren), n (%)	121 (76.1)
Consumption of alcohol, n (%)	78 (49.1)
Consumption of cigarettes	20 (12.6)
Special form of nutrition	23 (14.5)
History of depression	19 (11.9)
Highest educational level, n (%)	
Secondary school	31 (19.5)
High school diploma	2 (1.3)
Finished apprenticeship	74 (46.5)
University degree	35 (22)
n/a	17 (10.7)
Professional status, n (%)	
Unemployed	8 (5)
Employed	49 (30.8)
Self-employed	11 (6.9)
Retired	75 (47.2)
n/a	16 (10.1)
Religion, n (%)	
Cristian	104 (65.4)
Muslim	4 (2.5)
Jewish	1 (0.6)
Other religion	5 (3.1)
No religion	34 (21.4)
n/a	11 (6.9)
Support by family, n (%)	144 (90.6)
Postoperative data	
Intensive care stay, days	1 (1–1)
Hospitalization, days	10 (6–17.5)
Postoperative complications, n (%)	
No complications	28 (17.6)
Clavien-Dindo I	79 (49.7)
Clavien-Dindo II	10 (6.3)
Clavien-Dindo IIIa	21 (13.2)
Clavien-Dindo IIIb	1 (0.6)
Clavien-Dindo IVa	9 (5.7)
Clavien-Dindo IVb	5 (3.1)
Clavien-Dindo V	6 (3.8)

Data presented as median and interquartile range if not noted otherwise. ALPPS, associating liver partition with portal vein ligation for staged hepatectomy; *ALT, alanine aminotransferase; ASA, American society of anesthesiologists classification; AST, aspartate aminotransferase; BMI, body mass index; CCC, cholangiocellular carcinoma; CRLM, colo-rectal liver metastasis; FFP, fresh frozen plasma; FNH, focal nodular hyperplasia; GGT, gamma glutamyltransferase; HCC, hepatocellular carcinoma; INR, international normalized ratio; TSH, two stage hepatectom*.

### Univariate and multivariable analyses

Univariate and multivariable linear regression were conducted for all 8 domains and the two summary scales of SF-36. All univariate analyses are displayed in detail in Table 3. The statistically significant parameters were further transferred to multivariable analyses to identify independent associations.

For the PF domain, independent predictors included the presence of CVD (B = −8.1; *p* = 0.044), neoadjuvant therapy (B = 8.13; *p* = 0.039), Prothrombin time (B = 0.33; *p* = 0.005), hemoglobin level (B = 3.11; *p* = 0.009), history of depression (B = −17.4; *p* = 0.003), and professional status (retired/unemployed) (B = −11.14; *p* = 0.006). In the RP domain, neoadjuvant therapy (B = 20.75; *p* = 0.020), hemoglobin level (B = 9.48; *p* = 0.001), and CVD (B = −13.42; *p* = 0.036) were significant predictors. Regarding the BP domain, neoadjuvant therapy (B = 14.05; p = 0.003) and the presence of children (B = 15.06; *p* = 0.014) were associated with higher scores, while a history of depression (B = −14.93; *p* = 0.032) was the only negative predictor. In the GH domain, both history of depression (B = −15.01; *p* = 0.001) and being retired or unemployed (B = −7.49; *p* = 0.010) were negatively associated.

For they VT domain, neoadjuvant chemotherapy (B = 11.32; *p* = 0.001) and higher hemoglobin levels (B = 3.84; p = 0.001) were positive predictors, whereas female sex (B = −19.94; *p* = 0.001), CVD (B = −6.8; *p* = 0.026), and history of depression (B = −19.94; *p* = 0.001) were negative predictors. In the SF domain, female sex (B = −12.83; *p* = 0.007) was the only independent negative predictor, while higher hemoglobin levels (B = 1.99; *p* = 0.048) and preoperative chemotherapy (B = 9.85; *p* = 0.034) had a positive impact. For the RE domain, negative associations were found for female sex (B = −16.57; *p* = 0.026), higher creatinine levels (B = −26.67; *p* = 0.012), and history of depression (B = −35.16; *p* = 0.001), whereas neoadjuvant chemotherapy was associated with improved scores (B = 15.17; *p* = 0.031). In the MH domain, neoadjuvant chemotherapy (B = 7.33; *p* = 0.023) showed a positive association, while female sex (B = −11.7; p = 0.001), higher creatinine levels (B = −15.45; p = 0.001), and history of depression (B = −15.7; p = 0.001) were negative predictors.

Regarding the PCS, higher prothrombin time (B = 0.098; *p* = 0.046), hemoglobin levels (B = 1.33; *p* = 0.008), and neoadjuvant chemotherapy (B = 4.46; *p* = 0.007) were associated with higher scores, whereas being retired/unemployed (B = −3.68; p = 0.026) correlated with lower scores. For MCS, female sex (B = −8.55; p = 0.001), higher creatinine levels (B = −9.44; p = 0.001), and a history of depression (B = −10.06; p = 0.001) were significantly associated with decreased scores.

Considering those analyses, neoadjuvant conducted chemotherapy was the most frequent statistically significant parameter for QoL-impact (8/10 categories), followed by history of depression (7/10 categories) and female sex as well as higher hemoglobin levels (5/10 categories, respectively). R^2^ was assessed for multivariable analyses of QoL categories, respectively. The mean value for all 10 categories was 0.23. The highest values were 0.36 in VT category, followed by PF with value of 0.29 and MCS with 0.28. Lower R^2^ < 0.2 were observed in the PCS category (0.16) as well as in the BP and the GH categories with a value of 0.13, respectively. A detailed overview of multivariable analyses can be inferred from Table 4.

### Comparison of QoL

Except from the BP domain, the assessed cohort has a significant worse score in every domain and scale compared to the general German population [12]. For example, the median PCS and MCS were significantly lower with 42.93 and 42.73, compared to 48.36 and 50.87 in the general German population. Detailed descriptions can be inferred from Table 2.

**Table 2:** QoL in comparison with the 1998 German general population by SF-36.

Categories of SF 36	Assessed cohort (*n* = 159)	German population^a^ (*n* = 6964)	*p*-Value
PF	67.55 ± 26.11	85.41 ± 20.65	**<0.001**
RP	41.35 ± 41	82.36 ± 32.65	**<0.001**
BP	69.59 ± 29.8	67.38 ± 25.87	0.289
GH	54.76 ± 18.07	66.42 ± 18.16	**<0.001**
VT	50.41 ± 21.63	60.02 ± 17.84	**<0.001**
SF	63.92 ± 29.4	86.38 ± 19.92	**<0.001**
RE	52.2 ± 45.71	89.11 ± 26.69	**<0.001**
MH	62.19 ± 21.46	72.46 ± 16.69	**<0.001**
PCS	42.93 ± 10.28	48.36 ± 9.42	**<0.001**
MCS	42.73 ± 12.4	50.87 ± 8.82	**<0.001**

Data are presented in mean values with standard deviation. Statistically significant parameters are presented in bold brackets.

a Reference values are derived from the 1998 German Federal Health Survey (n = 6964) [12], BP, Bodily Pain; GH, General Health; MCS, Mental Component Summary; MH, Mental Health; PCS, Physical Component Summary; PF, Physical Functioning; RE, Role Emotional; RP, Role Physical; SF, Social Functioning; VT, Vitality.

**Table 3:** Univariate analysis for influential QoL parameters.

	PF	RP	BP	GH	VT	SF	RE	MH	PCS	MCS
Demographics										
Sex (male = 1); p	0.447	0.083	0.05	0.308	**0.001**	**0.001**	**0.014**	**0.001**	0.565	**0.001**
r	−0.61	−0.14	−0.16	−0.08	−0.29	−0.25	−0.19	−0.26	−0.05	−0.3
Age, years; p	**0.004**	0.083	0.314	0.312	0.398	0.774	0.091	0.966	0.103	0.921
r	−0.23	−0.14	0.08	−0.08	−0.07	0.02	−0.13	−0.01	−0.13	−0.01
BMI, kg/m^2^; p	0.201	0.2	0.976	0.612	0.987	0.258	0.915	0.973	0.845	0.567
r	−0.1	0.1	−0.01	−0.04	−0.01	0.09	−0.01	−0.01	−0.02	0.05
ASA (I/II = 1); p	0.19	0.495	0.288	0.439	0.062	0.74	0.499	0.748	0.722	0.354
r	−0.11	0.05	0.09	−0.06	0.06	0.03	0.05	0.03	−0.03	0.07
Diabetes mellitus (no = 1); p	**0.005**	0.262	0.733	0.348	0.587	0.903	0.201	0.578	**0.05**	0.863
r	−0.22	−0.09	−0.03	−0.08	−0.04	−0.01	−0.1	−0.04	−0.16	−0.01
Cardiovascular disease (no = 1); p	**0.001**	**0.035**	0.472	0.121	**0.043**	0.249	**0.006**	0.095	**0.027**	0.075
r	−0.25	−0.17	−0.06	−0.12	−0.16	−0.09	−0.22	−0.13	−0.18	−0.14
Pulmonary disease (no = 1); p	0.073	0.591	0.927	0.767	0.571	0.913	0.436	0.834	0.754	0.862
r	−0.14	0.04	0.01	−0.02	−0.05	−0.01	−0.06	−0.02	−0.03	−0.01
Chronic kidney disease (no = 1); p	0.413	0.626	0.506	0.949	0.6	0.951	0.855	0.54	0.695	0.874
r	−0.07	−0.04	−0.05	0.01	−0.04	−0.01	−0.02	−0.05	−0.03	−0.01
Neoadj. chemotherapy (no = 1); p	**0.015**	**0.005**	**0.003**	0.064	**0.002**	**0.045**	**0.007**	**0.004**	**0.017**	**0.008**
r	0.19	0.22	0.23	0.15	0.24	0.16	0.21	0.23	0.19	0.21
Liver disease (benign = 1); p	0.532	0.998	0.27	0.893	0.535	0.519	0.796	0.109	0.778	0.234
r	−0.05	0.01	0.09	−0.01	0.05	0.05	−0.02	0.13	−0.02	0.1

Preoperative liver function										
AST, U/l; p	0.089	0.731	0.193	0.41	0.106	0.575	0.733	0.216	0.155	0.593
r	−0.14	−0.03	−0.1	−0.07	−0.13	−0.05	0.03	0.1	−0.11	−0.04
ALT, U/l; p	0.245	0.285	0.552	0.396	0.926	0.461	0.767	0.504	0.259	0.323
r	−0.09	−0.09	−0.05	0.07	0.01	0.06	0.02	0.05	−0.09	0.08
GGT, U/l; p	**0.02**	0.093	0.258	0.782	0.342	0.489	0.927	0.712	**0.031**	0.359
r	−0.19	−0.14	−0.09	−0.02	−0.08	0.06	−0.01	0.03	−0.17	0.07
Bilirubin, mg; p /dl	0.112	0.305	0.16	0.854	0.613	0.67	0.717	0.974	0.108	0.67
r	−0.13	−0.08	−0.11	−0.02	−0.04	−0.03	−0.03	0.01	−0.13	0.03
Platelet count, /nl; p	0.769	**0.008**	0.42	0.841	**0.013**	0.361	0.172	0.15	0.366	0.099
r	−0.02	−0.21	−0.06	−0.02	−0.2	−0.07	−0.11	−0.12	−0.07	−0.13
INR; p	**0.003**	**0.016**	0.126	0.081	0.067	0.389	0.055	0.035	**0.026**	0.146
r	−0.23	−0.19	−0.12	−0.14	−0.15	−0.07	−0.15	−0.17	−0.18	−0.12
Quick, %; p	**0.001**	**0.022**	0.467	0.287	0.129	0.951	0.076	0.301	**0.02**	0.542
r	0.26	0.18	0.06	0.09	0.12	0.01	0.14	0.08	0.18	0.05
Creatinine, mg/dl; p	0.174	0.68	0.981	0.123	0.108	0.535	**0.002**	**0.013**	0.839	**0.01**
r	−0.11	−0.03	0.01	−0.12	−0.13	−0.05	−0.24	−0.2	0.02	−0.21
Hemoglobin, g/dl; p	**0.001**	**0.001**	0.137	0.478	**0.001**	**0.01**	**0.035**	**0.022**	**0.003**	**0.025**
r	0.26	0.34	0.12	0.06	0.32	0.2	0.17	0.18	0.24	0.18

Social status										
Marital status (no partnership = 1); p	0.294	0.462	0.751	0.923	0.11	0.875	0.764	0.074	0.807	0.397
r	0.09	−0.06	0.03	−0.01	0.13	0.01	0.03	0.15	−0.02	0.07
Child(ren) (no = 1); p	0.12	0.931	**0.032**	0.44	0.353	0.502	0.837	0.402	0.163	0.754
r	0.13	0.01	0.18	0.06	0.08	0.06	0.02	0.07	0.12	0.03
Religion (no = 1)	0.282	0.676	0.572	0.582	0.428	0.554	0.42	0.585	0.533	0.527
r	−0.09	−0.04	−0.05	−0.05	−0.07	−0.05	−0.07	−0.05	−0.05	−0.05
Consumption of alcohol (no = 1); p	0.6	0.419	0.07	0.693	0.139	0.058	0.237	0.415	0.514	0.159
r	0.04	0.07	0.15	0.03	0.12	0.16	0.1	0.07	0.05	0.12
Consumption of cigarettes (no = 1);p	0.585	0.979	0.385	0.754	0.767	0.944	0.695	0.593	0.456	0.746
r	0.05	−0.01	0.07	0.03	0.03	0.01	0.03	0.04	0.06	0.03
Special form of nutrition (no = 1); p	0.306	0.768	0.621	0.897	0.235	0.2	0.267	0.064	0.534	0.051
r	0.05	−0.02	−0.04	−0.01	−0.1	−0.11	−0.09	−0.15	0.05	−0.16
History of depression (no = 1); p	**0.003**	0.142	**0.028**	**0.001**	**0.001**	**0.008**	**0.001**	**0.001**	0.069	**0.001**
r	−0.24	−0.12	−0.18	−0.3	−0.38	−0.22	−0.33	−0.34	−0.15	−0.36
Educational level (lower = 1); p	0.286	0.38	0.782	0.859	0.38	0.854	0.264	0.483	0.848	0.639
r	0.09	0.07	−0.02	−0.02	−0.08	−0.02	0.09	0.06	−0.02	0.04
Professional level (unemployed/retired = 1); p	**0.001**	**0.032**	0.376	**0.011**	**0.041**	0.137	0.097	0.101	**0.004**	0.259
r	−0.33	−0.18	−0.08	−0.21	−0.17	−0.13	−0.14	−0.14	−0.24	−0.1
Support by family (no = 1); p	0.187	0.156	0.844	0.33	0.377	0.438	0.477	0.806	0.744	0.838
r	0.11	−0.12	−0.02	−0.08	−0.07	−0.06	0.06	−0.02	−0.03	−0.02

Various parameters are associated QoL parameters. Statistically significant parameters are presented in bold brackets. ALT, alanine aminotransferase; ASA, American society of anesthesiologists classification; AST, aspartate aminotransferase; BMI, body mass index; Bp, Bodily pain; GGT, gamma glutamyltransferase; GH, General health; INR, international normalized ratio; MCS, Mental Component Summary; MH, Mental Health; p, *p*-value; PCS, physical Component Summary; PF, physical Functioning; r, linear correlation coefficient; RE, Role Emotional; RP, Role physical; SF, Social Functioning; VT, Vitality.

**Table 4:** Independent variables for decreased quality of life.

Categories of SF 36	Female Sex	Cardio-vascular disease	Absence of Neoadjuvant therapy	Reduced Prothrombin time	Renal disease	Anemia	Absence of children	History of depression	Employed	R^2^
PF		X	X	X		X		X	X	0.29
RP		X	X			X				0.21
BP			X				X	X		0.13
GH								X	X	0.13
VT	X	X	X			X		X		0.36
SF	X		X			X				0.25
RE	X		X		X			X		0.22
MH	X		X		X			X		0.26
PCS			X	X		X			X	0.16
MCS	X				X			X		0.28

Various parameters showed statistical significance in multivariable models for QoL scores. Statistically significant parameters are presented with X, respectively. BP, Bodily Pain; GH, General Health; MCS, Mental Component Summary; MH, Mental Health; PCS, Physical Component Summary; PF, Physical Functioning; RE, Role Emotional; RP, Role Physical; SF, Social Functioning; VT, Vitality.

## Discussion and conclusion

Over the past decades, QoL has emerged as a critical endpoint in clinical medicine. In this study, we aimed to evaluate preoperative QoL in a large, single-center European cohort undergoing liver resection, utilizing the SF-36 questionnaire. To our knowledge, this is the first study specifically investigating preoperative predictors of QoL in a European population of patients with hepatobiliary diseases scheduled for liver surgery. Our findings reveal a significantly reduced QoL in these patients compared to the general German population. Multivariable analysis further identified the absence of preoperative chemotherapy, history of depression, anemia, and female sex as the primary independent predictors of reduced preoperative QoL. Additionally, CVD, elevated prothrombin time and renal disease, the absence of children, and an employed or self-employed professional status were also associated with lower QoL scores in at least one domain.

The fact that anemia is adversely associated with certain QoL-categories (VT and PCS in particular) does not surprise considering of its pathophysiological aspects. As most of the cohort suffers from malignancy, cancer-related anemia is an aspect to mention in that context. This anemia variant has been discussed over the last decades and results from a complex interaction between tumor cells and immune system which causes shorter survival rates of red blood cells, impairs erythropoiesis, adequate production of erythropoietin as well as absolute/relative utilization of iron [13], [14]. Thus, therapeutic approaches comprise substitution of erythropoietic-stimulating agents and iron therapy but are also associated with potential severe side effects (e.g. thrombosis) and might delay surgery [15], [16]. Anemia is commonly identified as negative predictor of QoL in the last decades which emphasis the clinical need for better management and therapy of this condition [17].

Interestingly, our analyses repeatedly identified female sex as predictor for decreased QoL scores. Sex related differences in QoL have been examined before in studies of patients with various malignancies and yielding different results. In a newer Irish study of 232 cancer survivors with different malignancies female patients showed a higher tendence for lower scores in different QoL subscales (physical functioning, fatigue, pain) based on European Organization for Research and Treatment of Cancer QOL Questionnaire [18]. A few studies of patients with liver diseases examined sex differences in QoL, mainly in the postoperative setting after liver transplantation. For example, Bianco et al. could demonstrate in a cohort of 52 patients with HCV (26 male/26 female) that female patients had a better QoL regarding emotional state and mental health and lower QoL in terms of role functioning, bodily pain and physical activity one year after liver transplantation [19]. However, another polish study with 238 patients who underwent liver transplantation due to chronic/acute liver failure showed no differences in postoperative QoL regarding to sex [20]. A recent publication has also underscored the inconsistent use of the terms “sex” and “sex” in clinical trials, further complicating comparisons [21]. Notably, in our study, female sex was primarily associated with reduced scores in mental health–related QoL domains, which does not fully align with the findings of prior studies. This may offer novel insights into the complex relationship between sex and QoL in the specific context of preoperative hepatobiliary disease. At the same time, these associations may also reflect factors not captured in our dataset, such as differences in reporting behavior, greater willingness to disclose psychological distress or external family and social pressures in the immediate preoperative period. Clinically, this underscores the importance of providing targeted psychological support to female patients during the preoperative period. Importantly, our study is the first to clearly demonstrate a significant association of sex on preoperative QoL among candidates for liver surgery. However, as our study was not specifically designed to evaluate sex-based differences, these findings should be interpreted with caution and warrant further investigation in future studies.

Interestingly, patients who received neoadjuvant chemotherapy reported higher QoL scores in several domains. While chemotherapy is often associated with impaired QoL, the tailored nature of modern regimens may mitigate side effects and preserve physical function to enable surgery. These patients may also be more familiar with their disease and prognosis than individuals who have been diagnosed shortly before surgery potentially contributing to improved QoL. Supporting this idea, a German study with 128 patients showed indeed better postoperative QoL after liver resection for metastasis compared to other etiologies [22]. The authors argued similarly, although we have not compared QoL between liver metastases and other indications in our analysis. In contrast to the aforementioned paper, a systematic review by 2021 demonstrated a lower QoL after resection and chemotherapy in elder patients with metastasis of colorectal carcinoma (CRC) [23].

Another expectable finding in our study is the significant relation between history of depression and decreased QoL scores in almost all categories. In our cohort depression is slightly overrepresented (11.9%) compared to a general 12-month prevalence of 6% for major depressive disorders [24]. With regard to depressive disorders in malignant diseases, a 2023 meta-analysis showed that depression is generally not a risk factor for increased cancer incidence, with the exception of lung and other smoking-related cancers [25]. Although not all patients in our study had malignant disease, it is reasonable—based on this evidence—to assume that the increased prevalence of depression in our cohort may be attributable, at least in part, to the burden of hepatobiliary disease, rather than serving as a causative factor. Furthermore, another meta-analysis identified an elevated risk for depression following colorectal cancer (CRC) diagnosis, along with reduced QoL and increased mortality in patients with comorbid depression [26]. Since colorectal liver metastasis is the main etiology in our cohort, our findings are in line with the current literature and emphasize a close supportive care for those patients [27]. Emerging perioperative literature also indicates that preoperative depression and anxiety may be associated with adverse postoperative outcomes, including complications, length of stay, readmission, pain, recovery trajectories and functional decline [28], [29]. Although postoperative outcomes related to mood symptoms were not formally addressed in the present analysis, these data strengthen the rationale for preoperative psychological screening and targeted supportive interventions in patients scheduled for major hepatobiliary surgery.

Interestingly, except for higher prothrombin time in 2 out of 10 QoL-categories, liver function is not markedly associated with QoL in this cohort. In contrast, a study involving 472 patients with newly diagnosed HCC found strong associations between liver function and QoL at time of diagnosis using the EORTC QLQ-C30 and QLQ-HCC18, but HCC was a minor etiology in our study possibly explaining attenuated findings [30]. Furthermore, as all included patients were deemed operable, it is reasonable to assume that hepatic function was at least partially compensated in all cases, limiting variability in laboratory values and thus explaining the weaker correlations observed. Finally, it underlines a careful indication and patients´ preparation in clinical work-up.

Furthermore, we were able to demonstrate the presence of cardiovascular and renal disease as statistically significant predictors of worsened QoL across three respective domains. As expected, CVD primarily affected the physical domains. Notably, however, renal disease also impacted the MCS score, in addition to the other two domains. Recent literature has extensively described the negative impact of CVD on QoL [31]. Moreover, an association between reduced health-related QoL and the incidence of CVD has been established [32]. In the context of impaired renal function, improved QoL following kidney transplantation has been recognized for decades [33].

These findings highlight the importance of considering patients' pre-existing conditions during clinical assessment in detailed manner. This is further emphasized by the observation that a more general assessment of pre-existing illnesses by the ASA score did account for variations in QoL.

Another noteworthy finding of our study is the association between employment status—specifically being self-employed or employed—and reduced QoL across the three primary physical domains. When considered in conjunction with hepatobiliary disease, employment status may represent an additional physical stressor that should be taken into account during the preoperative evaluation. Finally, we identified the absence of children as a predictor of lower QoL specifically in the Bodily Pain (BP) domain. While this finding is not well-documented in the existing literature and lacks a clear explanation, it appears to be of less clinical relevance compared to the other predictors discussed.

While our study provides novel insights into the predictors of preoperative quality of life (QoL) in patients undergoing liver resection, several limitations should be considered when interpreting the findings. First, the study was conducted at a single high-volume European center, which may limit the generalizability of the results, as patient selection and preoperative assessment reflect the authors' specific institutional practices. Second, since our analysis deliberately focused on the preoperative period, we are unable to draw conclusions about changes in QoL over time, particularly in the postoperative phase. Third, the timing of questionnaire administration one day prior to surgery may have affected patient responses, as imminent surgery can increase situational anxiety, pain perception or emotional distress. The characteristics of the study population itself, including the high prevalence of cardiovascular comorbidities and the proportion of patients with a documented history of depression, may have influenced the observed preoperative QoL scores and should therefore be considered when interpreting the results. In addition, the SF-36 was administered only once, and we did not include specific validated screening tools for current anxiety or depressive symptoms, as history of depression was based on medical documentation and/or ongoing antidepressant treatment. Moreover, the comparative analysis used published German SF-36 reference values from the 1998 Federal Health Survey [10]. These reference data may differ from our cohort with respect to age, sex distribution, comorbidities and calendar period, and this potentially introduces bias that limits the strength of conclusions drawn from the comparison with the general population. Additionally, it is important to acknowledge a potential selection bias, as only patients deemed suitable for surgery were included. Individuals with severe comorbidities, advanced frailty, or significantly impaired performance status may not have been referred for surgical evaluation and, therefore, were not represented in our cohort.

In conclusion, our study provides a comprehensive assessment of preoperative QoL and its predictors in a well-characterized European cohort undergoing liver resection for hepatobiliary diseases. To our knowledge, this is the first study focusing on preoperative QoL predictors in this setting, as most prior research has centered on postoperative outcomes or was conducted in Asian populations with HCC. Overall, QoL in this cohort is markedly impaired. Key positive predictors include neoadjuvant chemotherapy and higher hemoglobin levels, whereas female sex and a history of depression are associated with significantly worse scores. Recognizing these factors may help identify patients at risk for impaired QoL, thereby guiding preoperative counseling and targeted supportive interventions. The role of anemia in particular warrants further investigation, as optimizing hemoglobin levels may represent a modifiable factor for improving preoperative QoL in this population.

## CRediT authorship contribution statement

**Carlos Constantin Otto:** Writing – original draft, Methodology, Investigation, Formal analysis, Conceptualization. **Anna Mantas:** Writing – review & editing, Formal analysis. **Alexander Genchev:** Writing – review & editing, Investigation. **Aleksandar V. Kargaliev:** Writing – review & editing, Investigation, Conceptualization. **Amal Mugdad:** Writing – review & editing, Investigation, Conceptualization. **Smiths Sengkwawoh Lueong:** Writing – review & editing, Investigation, Conceptualization. **Anna Ewa Cyrek:** Writing – review & editing, Investigation. **Florian W.R. Vondran:** Writing – review & editing, Formal analysis. **Ulf P. Neumann:** Writing – review & editing, Supervision, Project administration, Formal analysis. **Jan Bednarsch:** Writing – original draft, Supervision, Project administration, Methodology, Formal analysis, Conceptualization.

## Consent for publication

Not applicable.

## Ethics approval

This study was conducted in accordance with the requirements of the Institutional Review Board (EK177-21), current version of the Declaration of Helsinki and the good clinical practice guidelines (ICH-GCP). Informed consent was obtained from all included patients. The full official name of Institutional Review Board in Aachen is the Independent Ethics Committee of the Faculty of Medicine at RWTH Aachen University.

## Consent to publish declaration

Not applicable.

## Previous communications

None.

## Funding sources

This work was supported by the UMEA ACS Program of the University Duisburg-Essen (Jan Bednarsch).

## Declaration of competing interest

The authors declare that they have no known competing financial interests or personal relationships that could have appeared to influence the work reported in this paper.

## Data Availability

Dataset used for this study is available from the corresponding author upon reasonable request.

## References

[1] Nault J.C., Paradis V., Ronot M., Zucman-Rossi J. (2022). Benign liver tumours: understanding molecular physiology to adapt clinical management. Nat Rev Gastroenterol Hepatol.

[2] (2016). EASL Clinical Practice Guidelines on the management of benign liver tumours. J Hepatol.

[3] Slevin M.L. (1992). Quality of life: philosophical question or clinical reality?. Bmj.

[4] Chiche L., Adam J.P. (2013). Diagnosis and management of benign liver tumors. Semin Liver Dis.

[5] Verbesey J.E., Simpson M.A., Pomposelli J.J., Richman E., Bracken A.M., Garrigan K. (2005). Living donor adult liver transplantation: a longitudinal study of the donor’s quality of life. Am J Transplant.

[6] Thuluvath A.J., Peipert J., Berkowitz R., Siddiqui O., Whitehead B., Thomas A. (2021). Donor quality of life after living donor liver transplantation: a review of the literature. Dig Med Res.

[7] Karimi M., Brazier J. (2016). Health, health-related quality of life, and quality of life: what is the difference?. Pharmacoeconomics.

[8] Otto C.C., Mantas A., Heij L.R., Heise D., Dewulf M., Lang S.A. (2024). Preoperative predictors for non-resectability in perihilar cholangiocarcinoma. World J Surg Oncol.

[9] Mantas A., Liu D., Otto C.C., Heij L.R., Heise D., Bruners P. (2024). Time to surgery is not an oncological risk factor in patients with cholangiocarcinoma undergoing curative-intent liver surgery. Sci Rep.

[10] Bellach B.E.U., Radoschewski F. (2000). Der SF-36 im Bundes-Gesundheitssurvey Erste Ergebnisse und neue Fragen. Bundesgesundheitsblatt Gesundheitsforschung Gesundheitsschutz.

[11] Ware J.E., Sherbourne C.D. (1992). The MOS 36-item short-form health survey (SF-36). I. Conceptual framework and item selection. Med Care.

[12] Ellert U., Bellach B.M. (1999). The SF-36 in the Federal Health Survey—description of a current normal sample. Gesundheitswesen (Bundesverband der Arzte des Offentlichen Gesundheitsdienstes (Germany)).

[13] Birgegård G., Aapro M.S., Bokemeyer C., Dicato M., Drings P., Hornedo J. (2005). Cancer-related anemia: pathogenesis, prevalence and treatment. Oncology.

[14] Gilreath J.A., Stenehjem D.D., Rodgers G.M. (2014). Diagnosis and treatment of cancer-related anemia. Am J Hematol.

[15] Van Doren L., Steinheiser M., Boykin K., Taylor K.J., Menendez M., Auerbach M. (2024). Expert consensus guidelines: Intravenous iron uses, formulations, administration, and management of reactions. Am J Hematol.

[16] Bohlius J., Bohlke K., Castelli R., Djulbegovic B., Lustberg M.B., Martino M. (2019). Management of cancer-associated anemia with erythropoiesis-stimulating agents: ASCO/ASH clinical practice guideline update. J Clin Oncol.

[17] Cortesi E., Gascón P., Henry D., Littlewood T., Milroy R., Pronzato P. (2005). Standard of care for cancer-related anemia: improving hemoglobin levels and quality of life. Oncology.

[18] Keaver L., O’Callaghan N., O’Sullivan A., Quinn L., Loftus A., McHugh C.M. (2021). Female cancer survivors are more likely to be at high risk of malnutrition and meet the threshold for clinical importance for a number of quality of life subscales. J Hum Nutr Diet.

[19] Bianco T., Cillo U., Amodio P., Zanus G., Salari A., Neri D. (2013). Gender differences in the quality of life of patients with liver cirrhosis related to hepatitis C after liver transplantation. Blood Purif.

[20] Dąbrowska-Bender M., Michałowicz B., Pączek L. (2016). Assessment of the quality of life in patients after liver transplantation as an important part of treatment results. Transplant Proc.

[21] Göttgens I., Oertelt-Prigione S. (2025). Gender as a contextual factor in quality of life of cancer survivors: a literature review. Oncol Res Treat.

[22] Bruns H., Krätschmer K., Hinz U., Brechtel A., Keller M., Büchler M.W. (2010). Quality of life after curative liver resection: a single center analysis. World J Gastroenterol.

[23] Alabraba E., Gomez D. (2021). Systematic review of treatments for colorectal metastases in elderly patients to guide surveillance cessation following hepatic resection for colorectal liver metastases. Am J Clin Oncol.

[24] Malhi G.S., Mann J.J. (2018). Depression. Lancet.

[25] van Tuijl L.A., Basten M., Pan K.Y., Vermeulen R., Portengen L., de Graeff A. (2023). Depression, anxiety, and the risk of cancer: an individual participant data meta-analysis. Cancer.

[26] Cheng V., Oveisi N., McTaggart-Cowan H., Loree J.M., Murphy R.A., De Vera M.A. (2022). Colorectal cancer and onset of anxiety and depression: a systematic review and meta-analysis. Curr Oncol.

[27] Ho M., Ho J.W.C., Fong D.Y.T., Lee C.F., Macfarlane D.J., Cerin E. (2020). Effects of dietary and physical activity interventions on generic and cancer-specific health-related quality of life, anxiety, and depression in colorectal cancer survivors: a randomized controlled trial. J Cancer Surviv.

[28] Geoffrion R., Koenig N.A., Zheng M., Sinclair N., Brotto L.A., Lee T. (2021). Preoperative depression and anxiety impact on inpatient surgery outcomes: a prospective cohort study. Ann Surg Open.

[29] Shebl M.A., Toraih E., Shebl M., Tolba A.M., Ahmed P., Banga H.S. (2025). Preoperative anxiety and its impact on surgical outcomes: a systematic review and meta-analysis. J Clin Transl Sci.

[30] Li L., Mo F., Hui E.P., Chan S.L., Koh J., Tang N.L.S. (2019). The association of liver function and quality of life of patients with liver cancer. BMC Gastroenterol.

[31] Chatzinikolaou A., Tzikas S., Lavdaniti M. (2021). Assessment of quality of life in patients with cardiovascular disease using the SF-36, MacNew, and EQ-5D-5L questionnaires. Cureus.

[32] Pinheiro L.C., Reshetnyak E., Sterling M.R., Richman J.S., Kern L.M., Safford M.M. (2019). Using health-related quality of life to predict cardiovascular disease events. Qual Life Res.

[33] Jofré R., López-Gómez J.M., Moreno F., Sanz-Guajardo D., Valderrábano F. (1998). Changes in quality of life after renal transplantation. Am J Kidney Dis.

